# Axillary surgery in women with sentinel node-positive operable breast cancer: a systematic review with meta-analyses

**DOI:** 10.1186/s40064-016-1712-9

**Published:** 2016-01-27

**Authors:** Mia Schmidt-Hansen, Nathan Bromham, Elise Hasler, Malcolm W. Reed

**Affiliations:** National Collaborating Centre for Cancer, Park House, Greyfriars Road, Cardiff, CF10 3AF Wales, UK; Brighton and Sussex Medical School, University of Sussex, Brighton, BN1 9PX UK; Brighton and Sussex University Teaching Hospitals Trust, Brighton, UK

**Keywords:** Breast cancer, Axillary surgery, Radiotherapy, Operable, Node positive, Sentinel lymph node dissection

## Abstract

Traditionally, women with node-positive operable breast cancer have received complete axillary lymph node dissection (ALND), which is associated with significant morbidity, but recently less invasive alternatives have been explored. We conducted a systematic review of randomised controlled trials assessing alternative approaches to axillary surgery in patients with pathologically-confirmed sentinel node-positive operable breast cancer. We searched on 16/3/15 the Specialized Register of the Cochrane Breast Cancer group; CENTRAL; MEDLINE; PreMEDLINE; EMBASE; WHO International Clinical Trials Registry Portal; ClinicalTrials.gov; conference proceedings from ASCO and the San Antonio Breast Cancer meetings; checked reference lists and contacted authors to identify relevant studies. Double, independent study sifting, extraction, appraisal and summarising were undertaken using standard Cochrane Collaboration methodology. We included three studies (2020 patients) comparing ALND with sentinel lymph node dissection (SLND) to SLND alone, and two studies (1899 patients) comparing ALND to axillary radiotherapy (aRT). No differences in survival or recurrence were observed between ALND and SLND or aRT, but morbidity may have been increased in ALND, and all the results were subject to different biases, such as recruitment bias, performance bias, and outcome-reporting bias. Whilst it is encouraging that there appears to be no adverse effect on recurrence or survival, it will be appropriate to confirm these findings and provide additional data confirming quality of life effects and long term outcomes.

**Electronic supplementary material:**

The online version of this article (doi:10.1186/s40064-016-1712-9) contains supplementary material, which is available to authorized users.

## Background

Current NICE Guidance for patients treated in the United Kingdom National Health Service makes the following recommendations:

- Offer further axillary treatment to patients with early invasive breast cancer who:
  - have macrometastases or micrometastases shown in a sentinel lymph node.
  - have a preoperative ultrasound-guided needle biopsy with histologically proven metastatic cancer.
  The preferred technique is axillary lymph node dissection (ALND) because it gives additional staging information.
- Do not offer further axillary treatment to patients found to have only isolated tumour cells in their sentinel lymph nodes. These patients should be regarded as lymph node-negative (NICE 2009).

This guidance was last updated in 2009 and is currently under review. Since then a number of studies have evaluated whether all patients identified as having metastatic breast cancer in the axillary sentinel nodes require completion axillary lymph node dissection (ALND) and whether radiotherapy might be an effective alternative to ALND in patients where further treatment is recommended following the identification of a positive axillary node. Traditionally complete or partial excision of axillary lymph nodes was common practice in the surgical treatment of patients with early breast cancer regardless of the presence or absence of metastatic disease. This provided information on likely prognosis and guidance on the selection of appropriate adjuvant therapies, including chemotherapy and radiotherapy, following mastectomy. Concerns relating to the morbidity associated with ALND, particularly arm swelling (lymphoedema), shoulder stiffness and neuropathic pain, resulted in the development of targeted procedures (sentinel lymph node biopsy [SNLB], axillary node sampling) designed to stage the axilla, with ALND only recommended for those patients where positive evidence of metastatic disease was identified. These techniques were shown to be associated with less morbidity in the group undergoing SLNB alone without any clear adverse impact on overall survival or disease free survival (Bromham et al.: Axillary staging for operable primary breast cancer (Cochrane Review), submitted). However, for those patients with a positive SLNB a second procedure (ALND) was required in most cases although preoperative molecular assessment or other techniques such as imprint cytology have been utilised in some centres to facilitate completion axillary node clearance as a single procedure in those found to have metastatic spread to the sentinel node/s.

The increased use of molecular markers (e.g. HER2, Oestrogen and Progesterone receptor status) has resulted in a reduced reliance on numerical axillary node status for adjuvant therapy decision-making and resulted in the proposal that ALND may not be indicated in patients with (limited) axillary node disease and, similarly, the proposal that radiotherapy might be associated with fewer side effects and similar outcomes to ALND.

### Objectives

To assess in a systematic review conducted and reported according to the PRISMA guidelines (Moher et al. 2009) the benefits and harms of alternative approaches to axillary surgery (including omitting such surgery altogether) in terms of overall survival; disease-free survival; local, regional and distant recurrences; short-term adverse events; and long-term complications in patients with pathologically-confirmed sentinel node-positive operable breast cancer.

## Methods

We included randomised controlled trials in women with clinically-defined operable primary breast cancer with a positive sentinel lymph node, comparing the following interventions as part of the initial surgical treatment of early breast cancer: ALND versus no axillary surgery; and ALND versus axillary radiotherapy without ALND; and reporting the following outcomes: Overall survival; disease-free survival; disease control in the axilla; breast cancer recurrence; adverse events; long-term complications; and quality-of-life. For all studies involving full axillary surgery or axillary sampling, the number of nodes removed and method of node analysis was recorded where available, to indicate whether an adequate sampling or clearance procedure was performed.

The search strategy consisted of the following searches (see Additional file 1 for full search strategies):

1. The Specialized Register of the Cochrane Breast Cancer group on 16 March 2015. Details of the sources and search strategies used to populate this register are described in the Group’s module in The Cochrane Library (http://onlinelibrary.wiley.com/o/cochrane/clabout/articles/BREASTCA/frame.html) Studies coded as “AXILLARY NODE(S)”, “EARLY BREAST CANCER”, “LOCALLY ADVANCED BREAST CANCER”, “PSYCHOSOCIAL”, or “SURGERY” on the specialised register has been extracted for consideration;
2. The Cochrane Central Register of Controlled Trials (CENTRAL) (*The Cochrane Library*, issue 2 on 16 March 2015);
3. MEDLINE via OvidSP (2007–12 March 2015) PreMEDLINE via OvidSP (12 March 2015) and EMBASE via OvidSP (2002–12 March 2015). We used a validated filter to identify reports of randomised controlled trials in the initial search of MEDLINE (Lefebvre and Clarke 2001) and for the updated searches used the revised filter (Lefebvre et al. 2011); we used the Scottish Intercollegiate Guidelines Network RCT filter for Embase (http://www.sign.ac.uk/methodology/filters.html);
4. The World Health Organisation International Clinical Trials Registry Portal (WHO ICTRP) and ClinicalTrials.gov for prospectively registered and ongoing trials, both on 16 March 2015;
5. The conference proceedings from the American Society of Clinical Oncology (ASCO) 41st–50th Annual Meetings (2005–2014) via the Journal of Clinical Oncology (http://jco.ascopubs.org/site/meetings) and the conference proceedings from the San Antonio Breast Cancer (SABCS) 29th-37th Annual Symposium Meetings (2006–2014) via Cancer Research web site (http://cancerres.aacrjournals.org/), both on 12 March 2015;
6. The authors of included or ongoing trials were contacted by e-mail and asked if they knew of any relevant studies, but no further studies were identified. The reference lists of the included studies as well as published reviews were also checked for relevant studies.

Two authors independently screened the titles and abstracts of the records identified in the electronic searches, excluding all obviously not relevant studies, and examined the full text of potentially eligible trials. If required, and possible, additional information was sought from the principal investigator of any trial of uncertain eligibility. Any discrepancies in eligibility judgements were resolved by discussion between the authors.

Study data from each trial were extracted independently by two authors with any disagreements in data extraction resolved by discussion between the authors. The authors of included studies were contacted by e-mail and asked to share unpublished data from their trial and to clarify any details about their trial that were missing or unclear in the published reports.

We assessed the risk of bias in the included studies using the standard Cochrane Collaboration methods for randomised trials (Higgins et al. 2011). Selection bias (random sequence generation, allocation concealment) and reporting bias (selective reporting) were assessed at study level, whereas detection bias (blinding of outcome assessment) and attrition bias (incomplete outcome data) were assessed at outcome level. We did not assess detection bias for the outcome of survival because this in an objective outcome, and we also did not assess performance bias because blinding of either healthcare personnel or patients is not possible with the interventions under consideration in this review.

The study data were meta-analysed where possible. The meta-analysis of time-to-event outcomes in Review Manager 5.3 (Nordic Cochrane Centre 2014) uses ‘O-E’ and ‘V’ statistics or hazard ratios (HR) for each trial. If these were not reported in a given trial we calculated them from the available statistics, if possible, using the methods described in Tierney (Tierney et al. 2007). Heterogeneity in meta-analyses was assessed using the I^2^ statistic. If the I^2^ value was >50 % we did not pool the effect estimates but used the range of effects from the individual studies instead. Time-to-event outcomes, entered as ‘O–E and Variance’ outcomes, were statistically synthesised using a fixed-effect model and arranged so that HRs > 1 favoured the ALND group and HRs < 1 favoured the comparison group. Dichotomous outcomes were summarised as risk ratios (RR) and analysed using a fixed-effects model according to the Mantel–Haenszel method and arranged so that RRs < 1 favoured the ALND group and RRs > 1 favoured the comparison group. All analyses were conducted in Review Manager 5.3 (Nordic Cochrane Centre 2014). We included only the data available in trial reports or through contact with the trial authors. No data imputation was attempted.

## Results

The search identified 7436 unique records, of which 7273 were excluded based on the title and abstract while the full publications of 163 potentially relevant studies were examined. Of these, 5 trials reported in 13 publications met the inclusion criteria, two studies were still ongoing (comparing ALND to SLNB [NCT01796444 (Wang 2013), and ALND or axillary radiotherapy [aRT] + adjuvant treatment versus adjuvant treatment alone [POSNOC (Goyal 2014a, b)], respectively) while the remaining 147 records were excluded because they were: not a randomised trial (n = 20), ineligible population (n = 101), unclear intervention (n = 2) and ineligible intervention (n = 24); See also Additional file 2). The five included studies compared ALND with sentinel lymph node dissection (SLND) to SLND alone [ACOSOG Z0011 (Lucci et al. 2007; Olsen and McCall 2008; Giuliano et al. 2010, 2011); ATTRM-048-13-2000 (Sola et al. 2013); IBCSG-23-01 (Galimberti et al. 2011, 2012, 2013)], and ALND to aRT [AMAROS (Straver et al. 2010a, b; Donker et al. 2014); OTOASOR (Savolt et al. 2013a, b)]. See Tables 1 and 2 for summary study details and risk-of-bias levels, respectively, and Additional file 3 for full study details and risk-of-bias assessments.

**Table 1:** Characteristics of the included studies

	ATTRM-048-13-2000	IBCSG-23-01	ACOSOG Z0011	AMAROS	OTOASOR
Study years	2001-2008	2001-2010	1999-2004	2001-2010	2002-2009
Comparison	SLND + ALND v SLND	SLND + ALND v SLND	SLND + ALND v SLND	ALND v aRT	ALND v aRT
ALND intervention	Breast conservation therapy or mastectomy + SLND + ”complete ALND” but not otherwise specified	Surgical resection of primary tumour + SLND + ALND not otherwise specified	Breast conserving surgery + SLND + ALND consisting of removal of all level I and II nodes on affected side with at least 10 identified nodes per surgical specimen	Breast-conserving treatment (including whole-breast radiotherapy or mastectomy with/without radiotherapy to the chest wall) + ALND (level I and II; at least 10 nodes)	Breast-conserving surgery or mastectomy + ALND (level I and II; at least 6 nodes)
Comparison intervention	Breast conservation therapy or mastectomy + SLND	Surgical resection of primary tumour + SLND	Breast conserving surgery + SLND: After the blue or hot nodes were removed any remaining axillary nodes were palpated and removed as SLNs if suggestive of disease	Breast-conserving treatment (including whole-breast radiotherapy or mastectomy with/without radiotherapy to the chest wall) + aRT, including the contents of all three levels of the axilla and the medial part of the supraclavicular fossa; 25 fractions of 2 Gy	Breast-conserving surgery or mastectomy + aRT including the contents of all three levels of the axilla and the supraclavicular fossa; 25 fractions of 2 Gy
Country	Spain	Europe, South America, Australia	USA	Europe	Hungary
Number ALND	112	464	436	744	244
Number Comparison	121	467	420	681	230
Age ALDN (years)	Mean = 55.3 (range 29–75)	Median = 53 (range 28–81)	Median = 56 (range 24–92)	Median = 56 (IQR 48–64)	Mean = 54.7 (range 26–74)
Age comparison (years)	Mean = 53.2 (range 33–75)	Median = 54 (range 26–81)	Median = 54 (range 25–90)	Median = 55 (IQR 48–63)	Mean = 55.2 (range 27–74)
Follow up	Median = 5.17 (range 2–9.17) years	Median = 5 (IQR 3.6–7.3) years	Median = 6.3 (IQR 5.2–7.7) years	Median = 6.1 (IQR 4.1–8) years	Mean = 41.9–42.3 months
Radiotherapy	Total breast (not axillary) both arms	Intraoperative with/without conventional post-operative RT: SLND alone: N = 410; ALND: N = 413	Whole breast RT. Some patients also received RT to the supraclavicular area (total N = 89).	Adjuvant aRT after ALND when ≥ 4 positive nodes were found. Adjuvant RT received to breast/chest wall/internal mammary chain: aRT: N = 546/51/65; ALND: N = 597/34/72.	ALND: Postoperative RT to the regional nodes when ≥ 4 positive nodes (pN2a-3a) or 1-3 positive nodes (pN1a) with other high-risk characteristics. 232 patients received RT to the breast/chest wall, 76 patients received RT to the axillary/supraclavicular nodes. aRT: 208 patients received RT to the breast/chest wall, 230 patients received RT to the axillary/supraclavicular nodes.
Chemo-therapy	41 ALND; 42 SLND alone	33 SLND alone; 42 ALND	243 ALND; 253 SLND alone	Yes according to local GLs	190 ALND; 159 aRT
Hormone therapy	10 ALND; 7 SLND alone	315 Surgery alone; 292 ALND	195 ALND; 203 SLND alone	Yes according to local GLs	213 ALND; 204 aRT
Both chemo- and hormone therapy	51 ALND; 65 SLND alone	103 Surgery alone; 107 ALND	Not reported	Not reported	159 ALND; 133 aRT
Baseline differences	Detection by palpation more in ALND	Appear comparable	Appear comparable	Appear comparable	More pT2-3 tumours in ALND
Intention-to-treat analyses	2 SLND alone and 4 ALND patients lost to follow up and not included in analyses	Yes for survival and disease-free survival. For the long term adverse events data were analysed per protocol	Yes for overall survival and recurrence	Yes	Unclear
Notes	14/247 randomised patients dropped out	Non-inferiority trial; closed early	Closed early	Non-inferiority trial	Equivalence trial

*SLND* sentinel lymph node dissection, *ALND* axillary lymph node dissection, *aRT* axillary radiotherapy, *RT* radiotherapy, *IQR* inter-quartile range

**Table 2:** Risk of bias in the included studies

	ATTRM-048-13-2000	IBCSG-23-01	ACOSOG Z0011	AMAROS	OTOASOR
Random sequence generation (selection bias)	Unclear	Low	Low	Low	Unclear
Allocation concealment (selection bias)	Unclear	Low	Low	Low	Unclear
Blinding of outcome assessment (detection bias): Disease control in the axilla	Unclear	High—no blinding	Unclear	High—no blinding	Unclear
Blinding of outcome assessment (detection bias): Breast cancer recurrence	Unclear	High—no blinding	Unclear	High—no blinding	Unclear
Blinding of outcome assessment (detection bias): Short term adverse events	Outcome not reported	High—no blinding	Unclear	Outcome not reported	Unclear
Blinding of outcome assessment (detection bias): Long term adverse events	Outcome not reported	High—no blinding	Unclear	High—no blinding	Unclear
Incomplete outcome data (attrition bias): Survival	Low	Low	Low	Low	Low
Incomplete outcome data (attrition bias): Disease control in the axilla	Low	Low	Low	Low	Unclear—data not reported in sufficient detail to be able to ascertain whether all patients are included
Incomplete outcome data (attrition bias): Breast cancer recurrence	Low	Low	Low	Low	Low
Incomplete outcome data (attrition bias): Short term adverse events	Outcome not reported	Unclear—denominator not reported	Unclear—data reported at 30 days for 371/411 ALND and 373/399 SLND + ALND	Outcome not reported	Outcome not reported
Incomplete outcome data (attrition bias): Long term complications	Outcome not reported	Unclear—14 patients allocated to surgery alone received ALND and 17 patients allocated to ALND did not receive ALND; all excluded from the analyses	High—data missing from progressively larger proportions of patients as follow up progressed; possibly more pronounced in the SLND group. Outcome data reported at 1 year for 242/411 ALND and 226/399 SLND +ALND	High—data available from 655/744 ALND and 586/681 aRT patients at baseline; progressively higher rates of missing data at 1, 3 and 5 years for lympoedema. Unclear how much data were available for shoulder mobility	Outcome not reported
Selective reporting (reporting bias)	High—adverse events not reported	Low	Low	High—only lymphedema and should mobility reported as morbidity outcomes	High—morbidity not reported

*SLND* sentinel lymph node dissection, *ALND* axillary lymph node dissection, *aRT* axillary radiotherapy

### ALND with SLND versus SLND

**Fig. 1:**
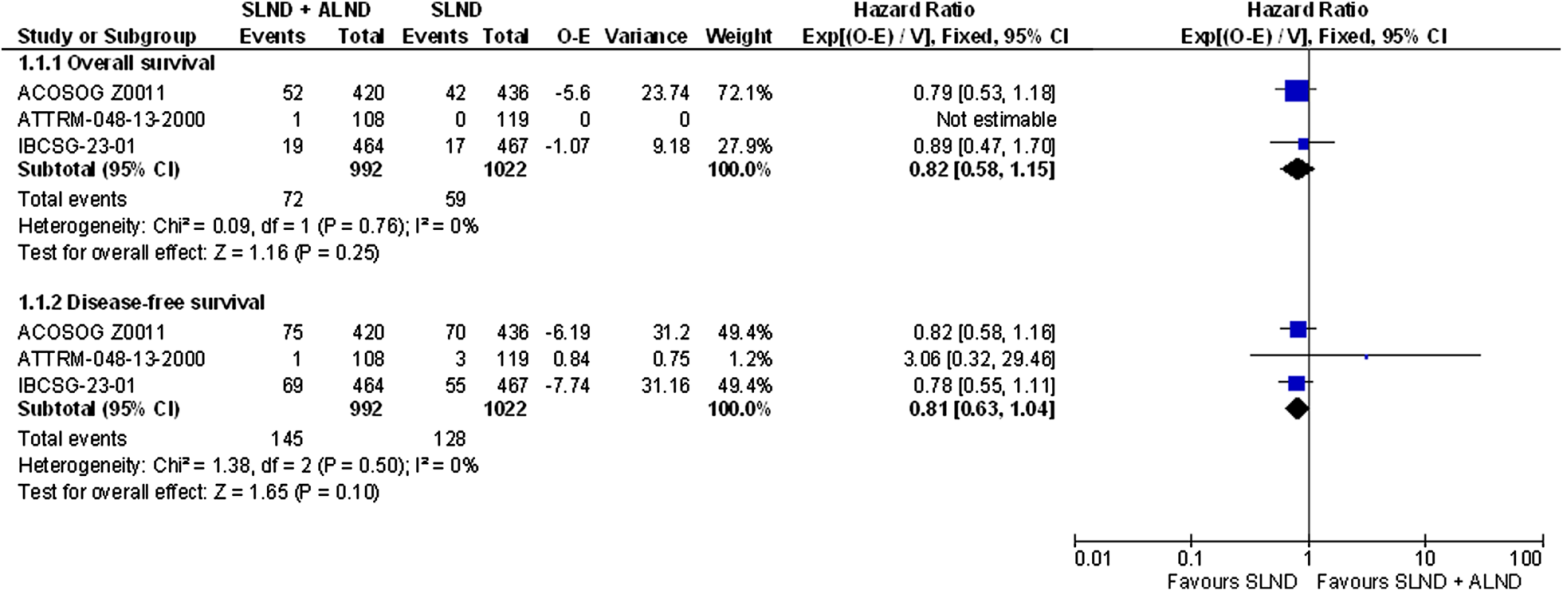
Overall survival and disease-free survival in the studies comparing SLND + ALND to SLND alone

Figure 1 shows that neither overall survival (summary HR = 0.82, 95 % CI 0.58–1.15; p = 0.25; I^2^ = 0 %) nor disease-free survival (summary HR = 0.81, 95 % CI 0.63–1.04; p = 0.1; I^2^ = 0 %) differed between the SLND + ALND and SLND treatment groups overall or in any of the trials.

**Fig. 2:**
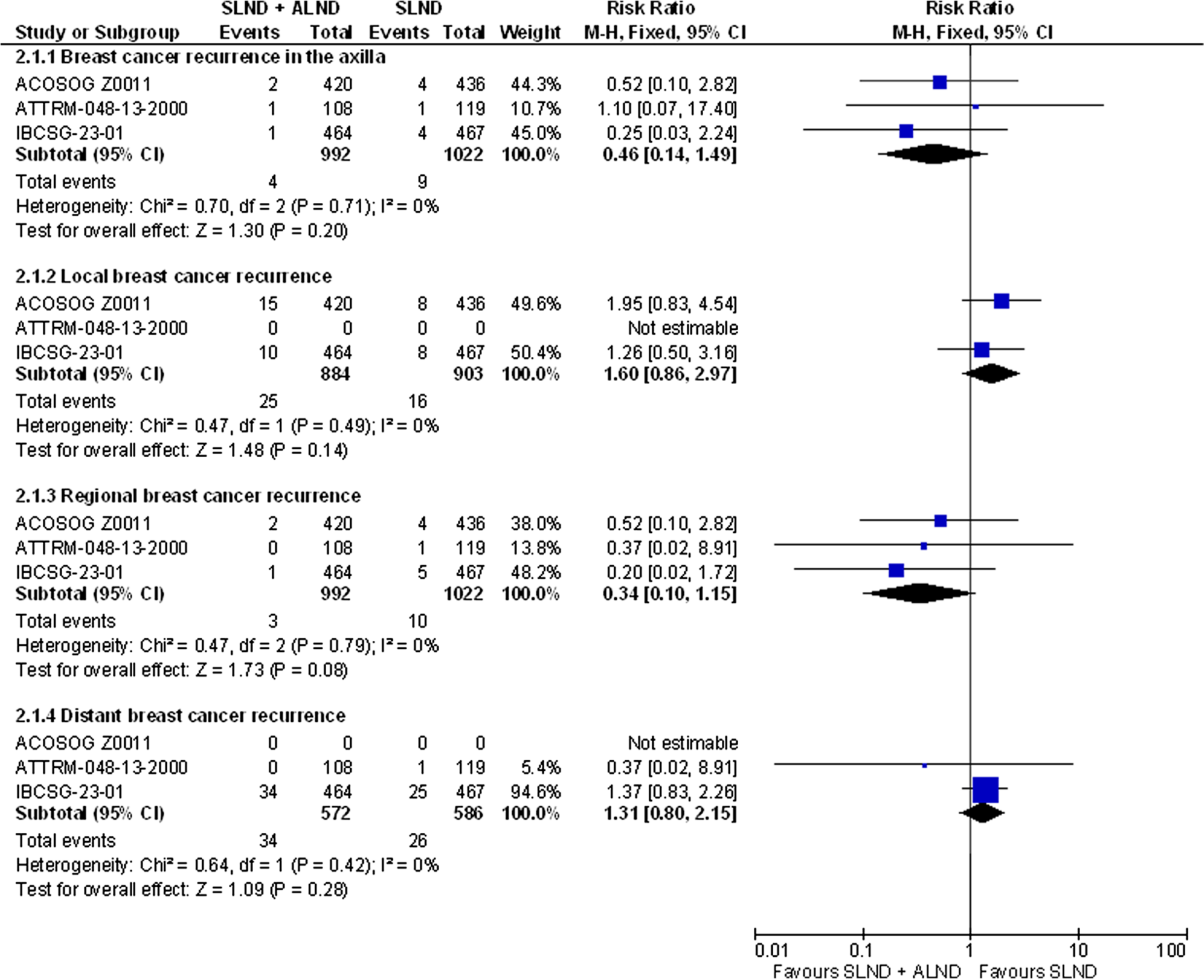
Breast cancer recurrence in the studies comparing SLND + ALND to SLND alone. Please note the following regarding the data included for ACOSOG ZOO11 for regional breast cancer recurrence: Regional recurrence defined as recurrence in the axillary, supraclavicular or internal mammary nodes. The authors only report local recurrence, axillary recurrence and locoregional recurrence. We have subtracted the local recurrence data from locoregional recurrence data to obtain the regional recurrence data, which is equal to the disease recurrence in the axilla data, suggesting that no patients recurred in the supraclavicular or internal mammary nodes, provided all these data only count each patient once. An entry of 0 in the total number of events column signifies that the study did not report this outcome

Meta-analysis of breast cancer recurrence as a dichotomous outcome, rather than as a time-to-event outcome, was undertaken as the data were not reported as time-to-event outcomes. However, the length of follow-up for these data was comparable between the trials (see Table 1). These analyses are illustrated in Fig. 2, which shows that axillary (summary RR = 0.46, 95 % CI 0.14–1.49; p = 0.2; I^2^ = 0 %), local (summary RR = 1.6, 95 % CI 0.86–2.97; p = 0.14; I^2^ = 0 %), regional (summary RR = 0.34, 95 % CI 0.1–1.15; p = 0.08; I^2^ = 0 %) and distant breast cancer recurrence (summary RR = 1.31, 95 % CI 0.8–2.15; p = 0.28; I^2^ = 0 %) did not differ between the treatment groups.

The ATTRM-048-13-2000 trial did not report on short-term adverse events or long term complications (Table 3) and is therefore at high risk of reporting bias for these outcomes. Inadequate details were reported on the selection of patients and the outcome assessment, which puts the results at risk of both patient selection bias and detection bias (Table 2). Moreover, at baseline the tumours were detected by palpation in more ALND than SLND patients. In IBCSG-23-01 the authors did not report inferential analyses of the short-term adverse events or long-term complications, but the rates of post-operative infection, sensory neuropathy (any, grade 3–4), lymphoedema (any, grade 3–4) and motor neuropathy were all numerically higher in the ALND group (Table 3). No blinding was undertaken of the outcome assessment, which means that the results are at high risk of detection bias. Moreover, it was unclear whether the results were subject to attrition bias for short-term adverse events and long-term complications (Table 2).

**Table 3:** Morbidity outcomes in the included studies

Comparison	SLND + ALND versus SLND alone			ALND versus aRT	
Study	ATTRM-048-13-2000	IBCSG-23-01	ACOSOG Z0011	AMAROS	OTOASOR
Short-term adverse events	Not reported	Post-operative infection: ALND: 1/464 SLND: 0/467	Wound infection: ALND: 31/373 SLND: 11/371; Axillary seromas: ALND: 53/373 SLND: 21/371; Axillary paresthesias: ALND: 174/373 SLND: 43/371; Objective lymphodema ^b^: ALND: 23/255 SLND: 17/272	Not reported	Not reported
Long-term complications	Not reported	Sensory neuropathy ^a^: *Any*: ALND: 82/447 SLND: 55/453 *Grade 3*–*4*: ALND: 1/447 SLND: 0/453 Lymphoedema ^a^: *Any*: ALND: 59/447 SLND: 15/453 *Grade 3*–*4*: ALND: 3/447 SLND: 0/453 Motor neuropathy ^a^: *Any*: ALND: 37/447 SLND: 13/453 *Grade 3*–*4*: ALND: 3/447 SLND: 1/453	Brachial plexus injury: *6* *months*: ALND: 5/406; SLND: 3/415; *12* *months*: ALND: 1/406; SLND: 0/415; Axillary paresthesias: *6* *months*: ALND: 146/335; SLND: 35/288; *12* *months*: ALND: 113/287; SLND: 24/268; Objective lymphoedema ^b^: *6* *months*: ALND: 29/270; SLND: 21/271; *12* *months*: ALND: 26/242; SLND: 14/226; Subjective lymphodema ^c^: *6* *months*: ALND: 27/327; SLND: 19/339; *12* *months*: ALND: 37/288; SLND: 12/268; >*12* *months*: ALND: 52/272; SLND: 14/253	Sign of lymphoedema ^d^: *Baseline:* ALND: 3/655; aRT: 0/586, p = 0.25; *12* *months*: ALND: 114/410; aRT: 62/410, p < 0.0001; *3* *years*: ALND: 84/373; aRT: 47/341, p = 0.003; *5* *years*: ALND: 76/328; aRT: 31/286, p < 0.0001; Arm circumference increase ≥ 10 % ^e^: *Baseline:* ALND: 33/655; aRT: 24/586, p = 0.5; *12* *months*: ALND: 32/410; aRT: 24/410, p = 0.332; *3* *years*: ALND: 38/373; aRT: 22/341, p = 0.08; *5* *years*: ALND: 43/328; aRT: 16/286, p = 0.0009; Shoulder mobility ^f^: No differences found in the range of motion in the four excursions at 1 (p = 0.29) or 5 years (p = 0.47).	Not reported
Quality of life	Not reported	Not reported	Not reported	No differences found^g^	Not reported

*ALND* axillary lymph node dissection, *SLND* sentinel lymph node dissection, *aRT* axillary radiotherapy

^a^The treating physician assessed and reported long-term surgical events (sensory neuropathy, lymphoedema, and motor neuropathy) at every follow-up visit (every 4 months from the date of randomisation for the first year, and every 6 months for years 2–5) on the basis of the National Cancer Institute Common Toxicity Criteria version 2. No more information reported

^b^Lympheoedema (objective): 2 cm or greater post-operative increase in the ipsilateral arm circumference (assessed by phycisian)

^c^Lympheoedema (subjective): according to patient self-report or physician diagnosis

^d^Any clinical sign of lymphoedema

^e^Arm circumference was measure 15 cm above the medial epicondyle (upper arms) and 15 cm below the medial epicondyle (lower arms). An increase in arm circumference of at least 10 % in the lower arm or the upper arm, or both, compared with the contralateral arm at the same timepoint was judged to be clinically significant lymphoedema

^f^The range of motion in both arms was measured in four excursions: abduction, adduction, anteversion, and retroversion and compared between arms. The four relative excursions were combined in a multivariate composite endpoint at 1 and 5 years

^g^Assessed using the EORTC quality-of-life questionnaire (EORTC-QLQ-C30; version 3) and breast cancer module (QLQ-BR23) using the pain, body image, and arm symptoms scales. The arm symptoms scale was composed of three items: pain in arm or shoulder, swollen arm or hand, and difficulties moving arm. Questionnaires were completed at baseline and at years 1, 2, 3, 5, and 10. All outcome data at 10 years subject to a future report

Inferential analyses of the rates of short-term adverse events were not presented in the ACOSOG Z0011 trial, but the rates of wound infection, axillary seromas, axillary paresthesias and objective lymphoma are all numerically higher in the group that received ALND. The same pattern of results was also observed for the long-term complications of brachial plexus injury, axillary paresthesias, and objective and subjective lymphoma at 6 and 12 months; and for subjective lymphoma at >12 months (Table 3). In this trial it was unclear whether outcome assessment was blinded, 30-day short-term adverse event data were not reported for all the patients, and the outcome data for long-term complications were missing for progressively larger proportions of patients in both treatment groups, but possibly more so for the SLND group. This in turn means that the results must be interpreted with some caution because they are at risk of detection bias for all outcomes and of attrition bias for the short-term adverse events outcome; and for the long-term complications the results are at high risk of attrition bias (Table 2). Moreover, all three trials randomised patients after the results of SLND were known, which puts these trials at risk of recruitment bias to the extent that patients perceived at higher risk (e.g., multiple micrometastatic foci) were not invited or chose not to take part in the studies. This is because any tendency not to recruit patients perceived to be at higher risk would influence the relative performance of the interventions in the direction that less extensive surgery (SLND) would appear relatively more beneficial because the patients who are more likely to benefit from more extensive surgery (ALND), that is, patients at higher risk, would not be part of the study population. This could mean that the results are only applicable to the patients seen in clinical practice who meet the inclusion criteria of these trials, but are also perceived to be at low risk.

### ALND versus aRT

In the AMAROS trial no differences in overall survival, disease-free survival, shoulder mobility or quality of life were observed between the groups that received ALND and aRT (Tables 3, 4). However, the rates of (any clinical sign of) lymphoedema were higher in the ALND group at 1, 3 and 5 years. When lymphoedema was defined as an arm circumference increase ≥10 %, the rates only differed significantly at 5 years (Table 3). The trial was open label and did not report short-term adverse events or long-term complications other than lymphoedema and shoulder mobility for which either progressively larger or unclear proportions of data were missing, respectively. The results are therefore at high risk of both detection bias (all outcomes), attrition bias (lymphoedema and shoulder mobility) and reporting bias (short-term adverse events and long term complications; Table 2).

**Table 4:** Overall survival, disease-free survival and breast cancer recurrence in the axilla in the studies comparing ALND and aRT

	Overall survival	Disease-free survival	Breast cancer recurrence in the axilla	
AMAROS	ALND: 71/744 aRT: 76/681; *5-year*: ALND: 93.3 % (95 % CI 91–95); aRT: 92.5 % (95 % CI 90–94.4); HR = 1.17 (95 % CI 0.85–1.62), p = 0.34	ALND: 124/744 aRT: 134/681; *5-year*: ALND: 86.9 % (95 % CI 84.1–89.3); aRT: 82.7 % (95 % CI 79.3–85.5); HR = 1.18 (95 % CI 0.93–1.51), p = 0.18	ALND: 4/744 aRT: 7/681; *5-year*: ALND: 0.43 % (95 % CI 0–0.92); aRT: 1.19 % (95 % CI 0.31–2.08)	None of the studies reported local, regional, locoregional or distant breast cancer recurrence
OTOASOR	No significant difference, although no overall rates reported	ALND: 94.3 % aRT: 97 %; non-significant	ALND: 0.82 % aRT: 1.3 %; non-significant	

The relative effects of treatments on time-to-event outcomes were reported so that HRs less than 1.0 favour the aRT arm and HRs greater than 1.0 favour the ALND arm

*ALND* axillary lymph node dissection, *aRT* axillary radiotherapy

The OTOASOR trial did also not find any significant differences between the treatment groups in overall survival, disease-free survival or axillary recurrence rates (Table 4), however, the OTOASOR trial did not report any morbidity outcomes, which puts the trial at risk of reporting bias. Moreover, very little information was reported about patient selection and allocation as well as about potential blinding of outcome assessment, which exposes the results to risk of both selection bias (all outcomes) and detection bias (all outcomes) to the extent that these were compromised (Table 2). At baseline, however, more ALND than aRT patients had pT2-3 tumours. On the other hand, both trials randomised patients before sentinel lymph node biopsy, which suggests that the study populations are representative of the risk spectrum of those patients seen in clinical practice that meet the inclusion criteria of these trials.

## Discussion

The evidence for ALND compared to other less invasive strategies for axillary treatment consisted of 5 studies including 3919 patients and reporting on 2 different comparisons: ALND versus aRT and SLND + ALND versus SLND. None of the included trials found a difference between the ALND groups and their respective comparison group in overall survival, disease-free survival or breast cancer recurrence. Two of the studies (IBCSG-23-01, ACOSOG Z0011) reported short-term adverse events and found that the rates were numerically higher in the ALND groups than in their respective comparison groups (neither study reported inferential analyses of these rates). Three of the studies (IBCSG-23-01, ACOSOG Z0011, AMAROS) reported long-term complications and found that lymphoedema tended to be higher in the ALND arms, either statistically significantly (AMAROS) or numerically (IBCSG-23-01, ACOSOG Z0011). Moreover, the rates of sensory neuropathy (IBCSG-23-01), motor neuropathy (IBCSG-23-01), brachial plexus injury (ACOSOG Z0011), and axillary paresthesias (ACOSOG Z0011) were also numerically higher in the ALND groups, although these results were also not analysed inferentially. Shoulder mobility and quality of life were not found to differ significantly between the treatment groups in the only study reporting these outcomes (AMAROS). These results were, however, subject to varying risks of a number of biases, not least detection bias, attrition bias, reporting bias and recruitment bias.

Although blinding of the patients and personnel is conceivably not feasible in the types of trials included in this review, blinding of outcome assessment may be undertaken in an effort to minimise the risk of detection bias. This bias is more likely to be at play for outcomes that are more subjective in evaluation rather than objective (e.g., survival). We therefore only considered this bias for breast cancer recurrence and morbidity. Detection bias in this context may lead to an overestimation of short-term adverse events and long-term complications in patients who received ALND. Similarly patients receiving less extensive axillary treatment may have been checked more carefully for breast cancer recurrence, because in both cases, the expected results would be of more morbidity in ALND and more recurrences in the less extensively treated axilla. The risk of attrition bias was particularly associated with the morbidity outcomes. This tended to be because these outcomes were assessed in a subset of the trial population. This subgroup of patients assessed for adverse events could be systematically different from the trial population as a whole, especially in the case of assessment for long-term complications when patients may have died or been too sick to participate. The morbidity outcomes were also at risk of reporting bias in the two studies that did not report morbidity at all (ATTRM, OTOASOR), while a third study only reported lymphoedema and shoulder mobility. Given the finding that none of the studies found any differences in overall or disease-free survival or in breast cancer recurrence, the assessment of treatment-associated morbidity arguably becomes less important when considering which treatment strategy to choose because it may be safe to assume that treatment-related morbidity will be less in less extensive treatments compared to ALND. However, it would still be preferable to be able to confirm the veracity of this assumption by being able to test it though appropriate analyses. Finally, we were unable to evaluate the risk of patient selection in two studies (ATTRM, OTOASOS) because not enough information was reported, which is of some concern because patient selection bias is a powerful bias that can affect the results markedly. Taken together, the risk of the different biases discussed above serves to compromise the validity of the results to the extent that they are at play and this must be borne in mind when considering the results.

Ram and colleagues (Ram et al. 2014) conducted a systematic review on SNLD alone versus SLND + ALND and included the same three RCTs included in the current review for that comparison, and unsurprisingly their conclusions are similar to ours for this comparison. Systematic reviews by Glechner et al. (2013) and Li et al. (2015), which included both RCTs and retrospective studies, also found no differences in overall survival, disease-free survival and recurrence between SLND alone compared to SLND + ALND, and higher rates of some adverse events associated with SLND + ALND, although Glechner et al. (2013) noted that a number of these results are subject to low event rates and/or the potential influence of different confounding variables and must be interpreted with caution. Moreover Li et al. (2015) treated ACOSOG ZOO11 as three RCTs, rather than one, which is potentially confusing and certainly at high risk of giving an inflated impression of the amount of evidence available. These reviews did not consider ALND versus aRT in patients with node-positive operable breast cancer, and we have found no other systematic reviews on that comparison either.

### Implications for practice

The studies described above have resulted in changes in guidelines and practice in some countries with implementation of the findings of the ACOSOG Z0011 study in patients who specifically meet the entrance criteria in many centres in the USA. This may be partly due to the inclusion of a significant number of patients with micrometastases in ACOSOG Z011 which are not now regarded as an indication for ALND or radiotherapy. Similarly there is increased use of axillary radiotherapy as an alternative to ALND following positive SLND in several European centres. However for the reasons identified above and in the following section these studies have not resulted in universal changes in practice and further data are required to confirm these results. Whilst it is encouraging that there appears to be no adverse effect on local, regional or distant recurrence or overall survival further evidence, particularly in those settings where evidence is lacking (e.g. mastectomy) will be appropriate to confirm these findings. At present many clinicians and multidisciplinary teams are interpreting and implementing these findings on a case by case basis in patients that strictly comply with the inclusion criteria of the relevant studies and whilst it is hoped this review will provide useful confirmation of the appropriateness of this practice, the provision of further data will be valuable not least in providing additional data confirming quality of life effects and long term outcomes.

### Implications for research

In this review, we only found 3 and 2 studies, respectively, evaluating each of the two target comparisons. Moreover, not all of these studies reported all the target outcomes. Quality-of-life was for example only reported by one of the included trials. We did however identify two ongoing trials that with time will contribute further data to this area (NCT01796444, POSNOC). These trials notwithstanding, the evidence base cannot be considered complete at this stage, and it would be preferable to see further well-designed and adequately powered studies conducted confirming the current results. Furthermore the increased stratification of the treatment of the axilla is being explored in further studies in both ‘low risk’ patients (e.g. no axillary staging) and ‘high risk’ patients (e.g. ALND followed by radiotherapy). It appears that research in the area of axillary node management in breast cancer will continue to drive the evolution from a ‘one size fits all’ approach to a more personalised evidence-based approach in future.

## Additional files

10.1186/s40064-016-1712-9 Full search strategies.
10.1186/s40064-016-1712-9 PRISMA diagram detailing the search results.
10.1186/s40064-016-1712-9 Full study characteristics of the included and ongoing studies.
