# Supplementary material for: Axillary surgery in women with sentinel node-positive operable breast cancer: a systematic review with meta-analyses
Source: Springerplus. 2016 Jan 27;5:85. doi: 10.1186/s40064-016-1712-9 (PMC4729721; doi:10.1186/s40064-016-1712-9)
Supplement: Supplementary file 1 — 10.1186/s40064-016-1712-9 Full search strategies. [file 40064_2016_1712_MOESM1_ESM.docx]

Supplementary Material for

**Title: Axillary surgery in women with sentinel node-positive operable breast cancer: A systematic review with meta-analyses**

**Authors:** Mia Schmidt-Hansen^1^, Nathan Bromham^1^, Elise Hasler^1^, Malcolm W Reed^2^

^1^National Collaborating Centre for Cancer, Park House, Greyfriars Road, Cardiff, CF10 3AF, Wales, UK
^2^ Dean, Brighton and Sussex Medical School, University of Sussex, Brighton, BN1 9PX, UK; and Honorary Consultant Surgeon, Brighton and Sussex University Teaching Hospitals Trust.

**Corresponding Author:** Mia Schmidt-Hansen^1^; email [Mia.Schmidt-Hansen@wales.nhs.uk](mailto:Mia.Schmidt-Hansen@wales.nhs.uk); tel: +44 2920 402910; fax: +44 2920 402911.

Additional file 1

**CENTRAL search strategy**

#1 MeSH descriptor: [Breast Neoplasms] explode all trees
#2 breast near cancer*
#3 breast near neoplasm*
#4 breast near carcinoma*
#5 breast near tumour*
#6 breast near tumor*
#7 #1 or #2 or #3 or #4 or #5 or #6
#8 MeSH descriptor: [Sentinel Lymph Node Biopsy] explode all trees
#9 sentinel lymph node biopsy or SLNB or SNB or SLN or (sentinel near node)
#10 MeSH descriptor: [Axilla] explode all trees
#11 axilla* near (surg* or sampl* or stag*)
#12 MeSH descriptor: [Neoplasm Staging] explode all trees
#13 MeSH descriptor: [Lymph Node Excision] explode all trees
#14 lymphadenectomy
#15 (block or lymph node or axillary) near dissection
#16 (block or lymph node or axillary) near clearance
#17 #8 or #9 or #10 or #11 or #12 or #13 or #14 or #15 or #16
#18 #7 and #17

**MEDLINE search strategy: Medline via OVIDSp**

1 exp Breast Neoplasms/

2 exp "Neoplasms, Ductal, Lobular, and Medullary"/

3 exp Fibrocystic Breast Disease/

4 or/1-3

5 exp Breast/

6 breast.tw.

7 5 or 6

8 (breast adj milk).ti,ab,sh.

9 (breast adj tender$).ti,ab,sh.

10 8 or 9

11 7 not 10

12 exp Neoplasms/

13 11 and 12

14 exp Lymphedema/

15 14 and 11

16 (breast adj25 neoplasm$).ti,ab,sh.

17 (breast adj25 cancer$).ti,ab,sh.

18 (breast adj25 tumour$).ti,ab,sh.

19 (breast adj25 tumor$).ti,ab,sh.

20 (breast adj25 carcinoma$).ti,ab,sh.

21 (breast adj25 adenocarcinoma$).ti,ab,sh.

22 (breast adj25 sarcoma$).ti,ab,sh.

23 (breast adj50 dcis).ti,ab,sh.

24 (breast adj25 ductal).ti,ab,sh.

25 (breast adj25 infiltrating).ti,ab,sh.

26 (breast adj25 intraductal).ti,ab,sh.

27 (breast adj25 lobular).ti,ab,sh.

28 (breast adj25 medullary).ti,ab,sh.

29 or/16-28

30 4 or 13 or 15 or 29

31 exp Mastectomy/

32 30 or 31

33 (mammary adj25 neoplasm$).ti,ab,sh.

34 (mammary adj25 cancer$).ti,ab,sh.

35 (mammary adj25 tumour$).ti,ab,sh.

36 (mammary adj25 tumor$).ti,ab,sh.

37 (mammary adj25 carcinoma$).ti,ab,sh.

38 (mammary adj25 adenocarcinoma$).ti,ab,sh.

39 (mammary adj25 sarcoma$).ti,ab,sh.

40 (mammary adj50 dcis).ti,ab,sh.

41 (mammary adj25 ductal).ti,ab,sh.

42 (mammary adj25 infiltrating).ti,ab,sh.

43 (mammary adj25 intraductal).ti,ab,sh.

44 (mammary adj25 lobular).ti,ab,sh.

45 (mammary adj25 medullary).ti,ab,sh.

46 or/33-45

47 32 or 46

48 exp Breast Self-Examination/

49 (breast adj25 self$).ti,ab,sh.

50 (breast adj25 screen$).ti,ab,sh.

51 exp Mammography/

52 or/47-51

53 mammograph$.tw.

54 53 and 11

55 52 or 54

56 randomized controlled trial.pt.

57 controlled clinical trial.pt.

58 randomized controlled trials.sh.

59 random allocation.sh.

60 double-blind method.sh.

61 single-blind method.sh.

62 or/56-61

63 clinical trial.pt.

64 exp Clinical Trials/

65 (clin$ adj25 trial$).ti,ab.

66 ((singl$ or doubl$ or trebl$ or tripl$) adj25 (blind$ or mask$)).ti,ab.

67 placebos.sh.

68 placebo$.ti,ab.

69 random$.ti,ab.

70 research design.sh.

71 or/63-70

72 62 or 71

73 55 and 72

74 (animals not humans).sh.

75 73 not 74

76 exp Sentinel Lymph Node Biopsy/

77 (sentinel adj2 node).mp.

78 (SN or SNB or SLN or SLNB).mp.

79 exp Axilla/

80 exp Neoplasm Staging/

81 exp Lymph Node Excision/

82 lymphadenectomy.mp.

83 (axill$ adj3 (surg$ or sampl$ or stag$)).mp.

84 ((block or lymph node or axillary) adj dissection).mp.

85 ((block or lymph node or axillary) adj clearance).mp.

86 or/76-85

87 75 and 86

**WHO ICTRP search strategy**

**Basic Search:**

1. Axillary staging for operable primary breast cancer
2. Breast cancer AND (axillary sampling OR axillary staging OR axillary surgery OR sentinel node biopsy OR sentinel lymph node biopsy)

**Advanced Search:**

1. Title: Axillary staging for operable primary breast cancer
Recruitment Status: ALL

2. Condition: Breast cancer
Intervention: axillary sampling OR axillary staging OR axillary surgery OR sentinel node biopsy OR sentinel lymph node biopsy
Recruitment Status: ALL

**ClinicalTrials.gov search strategy**

**Basic Search:**

1. Axillary staging for operable primary breast cancer
2. Breast cancer AND (axillary sampling OR axillary staging OR axillary surgery OR sentinel node biopsy OR sentinel lymph node biopsy)

**Advanced Search:**

1. Search Terms: Axillary staging for operable primary breast cancer
Recruitment: All studies
Study Results: All studies
Study Type: All studies
Gender: All studies

2. Conditions: Breast cancer
Interventions: axillary sampling OR axillary staging OR axillary surgery OR sentinel node biopsy OR sentinel lymph node biopsy
Recruitment: All studies
Study Results: All studies
Study Type: All studies
Gender: All studies
