# Supplementary material for: Axillary surgery in women with sentinel node-positive operable breast cancer: a systematic review with meta-analyses
Source: Springerplus. 2016 Jan 27;5:85. doi: 10.1186/s40064-016-1712-9 (PMC4729721; doi:10.1186/s40064-016-1712-9)
Supplement: Supplementary file 2 — 10.1186/s40064-016-1712-9 PRISMA diagram detailing the search results. [file 40064_2016_1712_MOESM2_ESM.docx]

Supplementary Material for

**Title: Axillary surgery in women with sentinel node-positive operable breast cancer: A systematic review with meta-analyses**

**Authors:** Mia Schmidt-Hansen^1^, Nathan Bromham^1^, Elise Hasler^1^, Malcolm W Reed^2^

^1^National Collaborating Centre for Cancer, Park House, Greyfriars Road, Cardiff, CF10 3AF, Wales, UK
^2^ Dean, Brighton and Sussex Medical School, University of Sussex, Brighton, BN1 9PX, UK; and Honorary Consultant Surgeon, Brighton and Sussex University Teaching Hospitals Trust.

**Corresponding Author:** Mia Schmidt-Hansen^1^; email [Mia.Schmidt-Hansen@wales.nhs.uk](mailto:Mia.Schmidt-Hansen@wales.nhs.uk); tel: +44 2920 402910; fax: +44 2920 402911.

Additional file 2

PRISMA diagram detailing the search results.

7436 records screened

7273 records excluded based on title/abstract

163 full text articles assessed for eligibility

150 records excluded:

- Ongoing studies: N = 2 (published in 3 articles);

- Not RCT: N = 20

- Ineligible population: N = 101;

Unclear intervention: N = 2;

Ineligible intervention: N = 24

5 studies (published in 13 articles) included in qualitative synthesis

3 studies (published in 8 articles) included in quantitative synthesis

8830 records identified through database searching

7436 records screened after duplicates removed

No additional records identified through other sources
