# Supplementary material for: Axillary surgery in women with sentinel node-positive operable breast cancer: a systematic review with meta-analyses
Source: Springerplus. 2016 Jan 27;5:85. doi: 10.1186/s40064-016-1712-9 (PMC4729721; doi:10.1186/s40064-016-1712-9)
Supplement: Supplementary file 3 — 10.1186/s40064-016-1712-9 Full study characteristics of the included and ongoing studies. [file 40064_2016_1712_MOESM3_ESM.docx]

Supplementary Material for

**Title: Axillary surgery in women with sentinel node-positive operable breast cancer: A systematic review with meta-analyses**

**Authors:** Mia Schmidt-Hansen^1^, Nathan Bromham^1^, Elise Hasler^1^, Malcolm W Reed^2^

^1^National Collaborating Centre for Cancer, Park House, Greyfriars Road, Cardiff, CF10 3AF, Wales, UK
^2^ Dean, Brighton and Sussex Medical School, University of Sussex, Brighton, BN1 9PX, UK; and Honorary Consultant Surgeon, Brighton and Sussex University Teaching Hospitals Trust.

**Corresponding Author:** Mia Schmidt-Hansen^1^; email [Mia.Schmidt-Hansen@wales.nhs.uk](mailto:Mia.Schmidt-Hansen@wales.nhs.uk); tel: +44 2920 402910; fax: +44 2920 402911.

Additional file 3

**Included studies**

**ACOSOG Z0011**

| **Methods** | Study design:  RCT  Country: USA  Study period: 1999-2004  Inclusion criteria: Patients who were ≥ 18 years and undergoing breast conservation therapy for clinical T1-2, N0, M0 breast cancer with 1-2 positive SLNs and ECOG status ≤ 2.  Exclusion criteria: No positive SLNs, time between patient’s first histologic diagnosis of invasive breast cancer and SLND > 60 days, breastfeeding, history of other malignancy within 5 years, bilateral breast cancer, multicentric disease, ≥ 3 positive SLNs, gross extracapsular invasion or matted nodes as SLND, medical contraindications to ALND or other risk factors precluding future treatment.  Length of follow up: Median = 6.3 (interquartile range 5.2-7.7) years for recurrence and survival. Adverse surgical effects were recorded at 30 days post-op, then every 6 months until year 3 post-op, and then annually. | |
| --- | --- | --- |
| **Participants** | 891 patients randomised: 445 to SLND + ALND and 446 to SLND only. 35 patients were excluded because they withdrew consent from the study Patient characteristics are reported for the remaining 856.  No. in trial arms (intent-to-treat sample): SLNB: N = 436 (N = 11 received ALND); SLND + ALND: N = 420 (N = 32 did not undergo ALND)  Age: SLNB: Median = 54 (range = 25-90) years; ALND: Median = 56 (range = 24-92) years.  Stage distribution: SLND: Clinical T1: N = 303; clinical T2: N =126; Missing: N = 7. ALND: Clinical T1: N = 284; clinical T2: N =134; Missing: N = 2.  Proportion node positive: SLNB: 386/415 (Missing N = 21); ALND: N = 339/343 (Missing N = 77).  Pathological type of breast cancer: SLNB: Inflitrating ductal: N = 356; infiltrating lobular: N = 36; other: N = 32; Missing: N = 12. ALND: Inflitrating ductal: N = 344; infiltrating lobular: N = 27; other: N = 45; Missing: N = 4. | |
| **Interventions** | All women had breast conserving surgery and SNLD (with 1 or 2 positive nodes) and were then randomised (either intraoperatively or post-operatively) to ALND or no ALND. ALND was done within 42 days of SLND. ANLD was defined as the removal of all level I and II nodes on the affected side with at least 10 identified nodes per surgical specimen. SLNs were identified using isosulfan blue, a radiopharmaceutical or both.  After the blue or hot nodes were removed any remaining axillary nodes were palpated and removed as SLNs if suggestive of disease. SLNs were positive if analysis of frozen sections, touch preparations or H&E stained permanent sections identified any metastasis. | |
| **Outcomes** | Surgical complications: wound infection, axillary seromas, axillary paraesthesia, lymphoedema and brachial plexus injury. | |
| **Axillary node surgery** | Minimum no. nodes to be removed according to protocol: Not reported.  Nodes removed SLND + ALND arm: Median = 17 (inter-quartile range 13-22) nodes per patient.  Nodes removed SNLD: Median = 2 (inter-quartile range 1-4) nodes per patient.  Method of node pathological analysis**:** Frozen sections, touch preparations or  hematoxylin-eosin (HE) stained permanent sections, but not immunohistochemistry. | |
| **Radiotherapy** | Both arms: All patients received whole-breast irradiation (4.5 – 5 Gy in fractions of  1.8-2 Gy/day 5 day/week) delivered via tangential fields with coplanar border. Some patients also received RT to the supraclavicular area (total N = 89).  RT same in all trial arms? Yes | |
| **Hormone and chemotherapy** | Adjuvant systemic therapy was delivered to 423 SLNB patients (chemotherapy N = 253, endocrine therapy N = 203) and to 403 ALND patients (chemotherapy N = 243, endocrine therapy N = 195). | |
| **Notes** | Of the 856 patients included, 56 SLND and 47 ALND patients were ineligible for the following reasons: Incorrect number of positive SNs (32 SLNDs, 16 ALNDs), SN positive by immunohistochemical staining only (4 SLNDs, 4 ALNDs), positive lumpectomy margins (7 SLNDs, 6 ALNDs), gross extracapsular extension in the SNs (7 SLNDs, 8 ALNDs) and other (6 SLNDs, 13 ALNDs).  Z0011 closed early due to slow accrual and lower death / recurrence rates than predicted.  Baseline differences? The paper states that the two groups of patients were balanced with respect to patient characteristics (see Table 1 page 3658 in Lucci et al 2007; and Table 1 page 429 in Guiliano et al 2010).  Intention to treat analyses? Yes for recurrence and survival. | |
| **Bias** | **Authors' judgement** | **Support for judgement** |
| Random sequence generation (selection bias) | Low risk | Random assignment by computer or interactive automatic telephone system. |
| Allocation concealment (selection bias) | Low risk | See cell above. |
| Blinding of outcome assessment (detection bias) Disease control in the axilla | Unclear risk | No details provided |
| Blinding of outcome assessment (detection bias) Breast cancer recurrence | Unclear risk | No details provided |
| Blinding of outcome assessment (detection bias) Short term adverse events | Unclear risk | No details provided |
| Blinding of outcome assessment (detection bias) Long term complications | Unclear risk | No details provided |
| Incomplete outcome data (attrition bias) Survival | Low risk | All patients are included in the analyses |
| Incomplete outcome data (attrition bias) Disease control in the axilla | Low risk | All patients appear to be included in the analyses |
| Incomplete outcome data (attrition bias) Breast cancer recurrence | Low risk | All patients appear to be included in the analyses |
| Incomplete outcome data (attrition bias) Short term adverse events | Unclear risk | Outcome data reported at 30 days for 371/411 and 373/399  in ALND and SLND +ALND arms respectively |
| Incomplete outcome data (attrition bias) Long term complications | High risk | Data missing from progressively larger proportions of patients as follow up progressed. A pattern that is possibly more pronounced in the SLND group. Outcome data reported at 1 year for 242/411 and 226/399  in ALND and SLND +ALND arms respectively. |
| Selective reporting (reporting bias) | Low risk | All major outcomes appear to be reported |

**AMAROS**

| **Methods** | Study design: RCT (multi-centre, non-inferiority)  Country: Europe  Study period: 2001-2010  Inclusion criteria: Patients with T1-2, primary, operable unifocal invasive breast cancer (5-30 mm) and clinically node negative ( with no palpable  lymphadenopathy). Bilateral breast cancer was not an exclusion criterion and there was no protocol-specified age limit. The protocol was amended in February 2008 to include tumours up to 5 cm diameter or multifocal  disease, or both. Sentinel nodes with only isolated tumour cells were also no longer regarded as sentinel node positive.  Exclusion criteria: Metastatic disease, previous neoadjuvant systemic treatment for the primary breast cancer, previous treatment of the axilla by surgery or radiotherapy, previous treatment of cancer (except basal cell carcinoma of the skin and in situ carcinoma of the cervix), or pregnancy.  Length of follow up: Median = 6.1 (IQR 4.1-8) years in sentinel node positive patients and 5.1 (IQR 3.9-6.3) years in sentinel node negative patients. | |
| --- | --- | --- |
| **Participants** | 2402 patients were randomly assigned to receive ALND and 2404 to receive aRT. 1425 patients were sentinel node positive; 744 ALND and and 681 aRT.  No. in trial arms: aRT: N = 681; ALND: N = 744  Age: aRT: Median = 55 (IQR = 48-63) years; ALND: Median = 56 (IQR = 48-64) years.  Stage distribution: Not reported, but Clinical tumour size was: aRT: Median = 18 (IQR = 13-23) mm; 0-2 cm: N = 533; 2-5 cm: N = 143; > 5 cm: N = 1; Missing: N = 4. ALND: Median = 17 (IQR = 13-22); 0-2 cm: N = 612; 2-5 cm: N = 132; > 5 cm: N = 0; Missing: N = 0.  Proportion node positive: All were SL+. aRT: Micrometastasis: N = 195; macrometastasis: N = 419; isolated tumour cells: N = 67. ALND: Micrometastasis: N = 215; macrometastasis: N = 442; isolated tumour cells: N = 87.  Pathological type of breast cancer: aRT: Infiltrating ductal: N = 515; infiltrating lobular: N =99; other: N = 66; missing: N = 1. ALND: Infiltrating ductal: N = 563; infiltrating lobular: N = 100; other: N =81; missing: N = 0. | |
| **Interventions** | Women were randomised before surgery and SLNB to the treatment they would receive if their sentinel lymph node biopsy (SLNB) proved positive. Women with negative SLNB received no additional treatment. Women with a positive lymph node received either axillary lymph node dissection (level I and II; at least 10 nodes; ALND) or axillary radiation therapy (including the contents of all three levels of the axilla and the medial part of the supraclavicular fossa; 25 fractions of 2 Gy; aRT).  Local treatment of the breast consisted of breast-conserving treatment (including whole-breast radiotherapy or mastectomy with/without radiotherapy to the chest wall).  Type of breast surgery; breast-conserving surgery/mastectomy/missing: aRT: N = 557/121/3; ALND: N = 609/127/8. | |
| **Outcomes** | 5-year axillary recurrence, axillary recurrence-free survival, disease-free survival, overall survival, shoulder mobility, lymphoedema, quality of life | |
| **Axillary node surgery** | Minimum no. nodes to be removed according to protocol: At least 1 sentinel lymph node. ALND: Dissection of at least anatomical levels I and II including at least 10 nodes.  Nodes removed aRT: Median = 2 (IQR 1-3) nodes (sentinel nodes)  Nodes removed ALND: Median = 2 (IQR 1-3) sentinel nodes + a median of 15 (IQR 12-20) additional nodes.  Method of node pathological analysis: “As a minimum requirement, three histological levels (500 micron distance) for each sentinel node were examined. On each level, two parallel sections were performed, one for immunohistochemistry and one for H&E staining. Immunohistochemical staining was required only when H&E staining was negative.” Tumour deposits were classified as isolated tumour cells (<0.2mm), micro-metastatis (0.2 to 2mm) or macro-metastasis (> 2mm). | |
| **Radiotherapy** | Both arms: Adjuvant axillary radiotherapy after axillary lymph node dissection was allowed when at least four positive nodes were found.  Adjuvant radiotherapy received to breast/chest wall/internal mammary chain: aRT: N =546/51/65; ALND: N = 597/34/72.  RT same in all trial arms? No | |
| **Hormone and chemotherapy** | Patients could also receive adjuvant systemic chemo/endocrine therapy according to local guidelines.  Systemic treatment received: Any/chemotherapy/hormonal therapy/immunotherapy: aRT: N =612/418/525/44; ALND: N = 666/453/585/45. | |
| **Notes** | 17 of the 4823 enrolled were excluded as they did not provide informed consent. In 132 patients the sentinel node could not be identified.  Baseline differences? The groups appear to be comparable at baseline.  Intention to treat analyses? Yes | |
| **Bias** | **Authors' judgement** | **Support for judgement** |
| Random sequence generation (selection bias) | Low risk | Patients were randomly assigned (1:1) by a computer-generated allocation schedule at the EORTC head quarters to axillary lymph node dissection or axillary radiotherapy  before sentinel node biopsy. Stratification was done by  institution using a minimisation method. |
| Allocation concealment (selection bias) | Low risk | See cell above |
| Blinding of outcome assessment (detection bias) Disease control in the axilla | High risk | The trial is described as “open-label”. |
| Blinding of outcome assessment (detection bias) Breast cancer recurrence | High risk | The trial is described as “open-label”. |
| Blinding of outcome assessment (detection bias) Short term adverse events | Unclear risk | Outcome not reported |
| Blinding of outcome assessment (detection bias) Long term complications | High risk | The trial is described as “open-label”. |
| Incomplete outcome data (attrition bias) Survival | Low risk | Data from all the patients were included in the analyses. |
| Incomplete outcome data (attrition bias) Disease control in the axilla | Low risk | Data from all the patients were included in the analyses. |
| Incomplete outcome data (attrition bias) Breast cancer recurrence | Low risk | Data from all the patients were included in the analyses. |
| Incomplete outcome data (attrition bias) Short term adverse events | Unclear risk | Outcome not reported. |
| Incomplete outcome data (attrition bias) Long term complications | High risk | Data available from 655/744 ALND and 586/681 aRT patients at baseline, and from the on progressively higher rates of missing data at 1, 3 and 5 years for lympoedema. Unclear how much data were available for shoulder mobility. |
| Selective reporting (reporting bias) | High risk | Only lymphoedema and shoulder mobility reported as morbidity outcomes. |

**AATRM-048-13-2000**

| **Methods** | Study design: RCT (multi-centre)  Country: Spain  Study period: 2001-2008  Inclusion criteria: Patients who were ≤ 75 years with newly diagnosed early (T < 3.5 cm, clinical N0, M0) stage breast cancer who had undergone breast conservation therapy or mastectomy as the primary treatment. All had micrometastatic (≥ 1 metastatic cell deposit no larger than 2 mm up until 2002 and then ≥ 1 metastatic cell deposit 0.2-2 mm) SN.  Exclusion criteria: Pregnant or breastfeeding women, age > 75 years, and ineligibility for follow up.  Length of follow up: Median clinical = 62 (range 24-107) months. | |
| --- | --- | --- |
| **Participants** | No. in trial arms: Observation: N = 121; ALND: N = 112  Age: Observation: Mean = 53.2 (range = 33-75) years; ALND: Mean = 55.3 (range = 29-75) years.  Stage distribution: Not reported, but Mean tumour size was: Observation: 1.78 (range = 0.1-3.5) cm. ALND: 1.57 (range = 0.15-3.5).  Proportion node positive: All were SL+. Observation: Other axillary nodes not evaluated; ALND: N = 15.  Pathological type of breast cancer: Observation: Ductal: N = 105; lobular: N = 6; other: N = 7; missing: N = 3. ALND: Ductal: N = 103; lobular: N = 4; other: N = 4; missing: N = 1. | |
| **Interventions** | Surgical excision as primary treatment + complete ALND (not otherwise specified) vs surgical excision as primary treatment + observation | |
| **Outcomes** | Disease-free survival, survival. | |
| **Axillary node surgery** | Minimum no. nodes to be removed according to protocol: Not reported.  Nodes removed Observation: NA  Nodes removed ALND: Not reported.  Method of node pathological analysis: Serial slices and immunohistochemical staining for cytokeratins with instructions to obtrain macroscopic tissue sections 1-2 mm thick, which were then sectioned at various levels and alternately stained with hematoxylin-eosin and anticytokeratin antibodies. Intraoperative diagnosis was optional. | |
| **Radiotherapy** | Both arms: Breast conserving therapy was followed by total breast radiotherapy, with care taken to avoid axillary radiation as much as possible. Thus only tangent (2-field) radiation was used; high tangents and a separate third axillary field were not allowed. Partial breast irradiation was not used in any case and mastectomy patients did not receive radiation (Observation: N = 8; ALND: N = 10).  RT same in all trial arms? Yes | |
| **Hormone and chemotherapy** | All patients received postoperative adjuvant systemic therapy (chemotherapy or hormone therapy according to the guidelines used at each centre). For the observation group 42 patients had chemotherapy, 7 patients had hormone therapy and 65 patients had both. For the ALND group 41 patients had chemotherapy, 10 patients had hormone therapy and 51 patients had both. | |
| **Notes** | Of the 247 patients randomised, 3 observation and 11 ALND patients dropped out after enrolment by personal choice  Baseline differences? The paper states that the two groups of patients were balanced with respect to patient characteristics with the exception that detection by palpation was more frequent in the ALND than on the observation group.  Intention to treat analyses? 2 Observation and 4 ALND patients were lost to follow up and not included in the analyses. No protocol violations reported. | |
| **Bias** | **Authors' judgement** | **Support for judgement** |
| Random sequence generation (selection bias) | Unclear risk | Method of random sequence generation not reported. |
| Allocation concealment (selection bias) | Unclear risk | Method of allocation concealment not reported. |
| Blinding of outcome assessment (detection bias) Disease control in the axilla | Unclear risk | No details reported |
| Blinding of outcome assessment (detection bias) Breast cancer recurrence | Unclear risk | No details reported |
| Blinding of outcome assessment (detection bias) Short term adverse events | Unclear risk | Outcome not reported |
| Blinding of outcome assessment (detection bias) Long term complications | Unclear risk | Outcome not reported |
| Incomplete outcome data (attrition bias) Survival | Low risk | 2 Observation and 4 ALND patients were lost to follow up and not included in the analyses. |
| Incomplete outcome data (attrition bias) Disease control in the axilla | Low risk | 2 Observation and 4 ALND patients were lost to follow up and not included in the analyses. |
| Incomplete outcome data (attrition bias) Breast cancer recurrence | Low risk | 2 Observation and 4 ALND patients were lost to follow up and not included in the analyses. |
| Incomplete outcome data (attrition bias) Short term adverse events | Unclear risk | Outcome not reported |
| Incomplete outcome data (attrition bias) Long term complications | Unclear risk | Outcome not reported |
| Selective reporting (reporting bias) | High risk | Adverse events not reported |

**IBCSG-23-01**

| **Methods** | Study design:  RCT (multi-centre, non-inferiority)  Country: Europe, South America and Australia  Study period:2001-2010  Inclusion criteria: Females of any age with clinical, mammographic, ultrasonographic, or pathological diagnosis of breast cancer, provided they had no previous or concomitant malignancy, pure ductal carcinoma in situ, previous systemic therapy for breast cancer, cancer chemoprevention treatment in the preceding year, distant metastases, palpable axillary nodes, or Paget’s disease without invasive cancer. In June 2006 the criteria for eligibility were broadened to include patients with one or more positive sentinel nodes (formerly only one); multicentric or multifocal tumours (formerly only unicentric); and largest lesion size of 5 cm or smaller (formerly ≤3 cm). Patients could be scheduled for mastectomy or conservative breast surgery. They were included in the trial and randomly assigned to treatment if, during or after surgical treatment for breast cancer, they were found to have a tumour of a maximum diameter of 5 cm or less by pathological measurement of the surgical specimen, and one or more micrometastatic foci (≤2 mm) in the sentinel nodes, but no macrometastatic disease. Isolated tumour cells were included within the definition of micrometastatic.  Exclusion criteria: Previous or concomitant malignancy, pure ductal carcinoma in situ, previous systemic therapy for breast cancer, cancer chemoprevention treatment in the preceding year, distant metastases, palpable axillary nodes, or Paget’s disease without invasive cancer. Pregnant or lactating women.  Length of follow up: Median = 5 (interquartile range = 3.6-7.3) years. | |
| --- | --- | --- |
| **Participants** | No. in trial arms: Surgery alone: N = 467; ALND: N = 464 (in addition to these patients, 3 randomised patients were excluded from the analyses as 2 had no data submitted due to no tumour found in the sentinel node and one patient withdrew consent).  Age: Surgery alone: Median = 54 (range = 26-81) years; ALND: Median = 53 (range = 28-81) years.  Stage distribution: Not reported, but tumour size was: Surgery alone: < 2 cm N = 322, 2-2.9 cm N = 112, ≥ 3 cm N = 28, unknown N = 5. ALND: < 2 cm N = 316, 2-2.9 cm N = 106, ≥ 3 cm N = 35, unknown N = 7.  Proportion node positive: All patients were sentinel node positive; N = 12 surgery alone patients and N = 59 ALND patients also had additional involved nodes.  Pathological type of breast cancer: Not reported, but oestrogen receptor status was: Surgery alone: Positive N = 425, negative N = 40, unknown N = 2; ALND: Positive N = 409, negative N = 51, unknown N = 4. | |
| **Interventions** | Surgical resection of primary tumour + ALND (not otherwise specified) versus surgical resection of the primary tumour without ALND | |
| **Outcomes** | Disease-free survival, overall survival, site of recurrence, short and long term surgical complications | |
| **Axillary node surgery** | Minimum no. nodes to be removed according to protocol: Not reported  Nodes removed ALND arm: Median = 21 (range = 1-44).  Nodes removed no axillary surgery: Median = 2 (range = 1-29).  Method of node pathological analysis**:** All sentinel nodes were entirely sectioned at 50–200 μm intervals and all sections (frozen or permanent) were examined with haematoxylin and eosin staining by pathologists at each participating centre. Cytokeratin immunostaining was used only when the presence of micrometastases was suspected, but not certain, or not determined, on haematoxylin and eosin-stained sections. | |
| **Radiotherapy** | Both arms: Patients either received conventional postoperative radiotherapy alone, in combination with intra-operative treatment or intraoperative treatment alone. Adjuvant radiotherapy consisted of one-shot intra-operative treatment with electrons (alone or in combination with postoperative radiotherapy) in 230 (27%) of patients who received breast-conserving surgery.  Surgery alone: 410/420 breast-conserving therapy patients received radiotherapy  ALND: 413/425 breast-conserving therapy patients received radiotherapy  RT same in all trial arms? Yes | |
| **Hormone and chemotherapy** | Both arms: Hormonal therapy alone was given to 315 surgery alone and 292 ALND patients, chemotherapy alone was given to 33 surgery alone and 42 ALND patients, and combinations of hormonal therapy and chemotherapy were given to 103 surgery alone and 107 ALND patients. | |
| **Notes** | Trial closed early after accrual of 934 / a projected 1960. 14 patients allocated to surgery alone received ALND and 17 patients allocated to ALND did not receive ALND.  Baseline differences? The baseline characteristics appear to be balanced according to randomly assigned treatment arm.  Intention to treat analyses? Yes for survival and disease-free survival. For the long term adverse events data were analysed per protocol. | |
| **Bias** | **Authors' judgement** | **Support for judgement** |
| Random sequence generation (selection bias) | Low risk | Computer generated - stratified by participating centre and menopausal status. |
| Allocation concealment (selection bias) | Low risk | Central allocation by computer. |
| Blinding of outcome assessment (detection bias) Disease control in the axilla | High risk | No blinding undertaken |
| Blinding of outcome assessment (detection bias) Breast cancer recurrence | High risk | No blinding undertaken |
| Blinding of outcome assessment (detection bias) Short term adverse events | High risk | No blinding undertaken |
| Blinding of outcome assessment (detection bias) Long term complications | High risk | No blinding undertaken |
| Incomplete outcome data (attrition bias) Survival | Low risk | All data appear to be included |
| Incomplete outcome data (attrition bias) Disease control in the axilla | Low risk | All data appear to be included |
| Incomplete outcome data (attrition bias) Breast cancer recurrence | Low risk | All data appear to be included |
| Incomplete outcome data (attrition bias) Short term adverse events | Unclear risk | Denominator not reported for short term adverse events Galimberti (2013) p. 302. |
| Incomplete outcome data (attrition bias) Long term complications | Unclear risk | 14 patients allocated to surgery alone received ALND and 17 patients allocated to ALND did not receive ALND. These patients were excluded from the analyses. |
| Selective reporting (reporting bias) | Low risk | All major outcomes appear to be reported |

**OTOASOR**

| **Methods** | Study design: RCT (single-centre, equivalence)  Country: Hungary  Study period: 2002-2009  Inclusion criteria: Women with primary invasive T1/2N0M0 breast tumours, clinically < 3 cm in diameter, no clinical suspicion of axillary lymph node involvement and no axillary lymphadenopathy.  Exclusion criteria: Age > 75 years; life expectancy without cancer < 5 years; inadequate performance status; noninfiltrating carcinoma or non-invasive malignancy (DCIS, LCIS); previous other malignancy; previous excision biopsy of the breast primary, chemotherapy or endocrine treatment; pregnancy; breast tumour > 3 cm; clinically evident metastatic involvement of the axilla or lack of axillary lymphatic drainage on isotope or blue stain.  Length of follow up: ALND: Mean =41.9 months; aRT: Mean = 42.3 months . | |
| --- | --- | --- |
| **Participants** | 1054 patients were randomly assigned to receive ALND and 1052 to receive aRT. 526 patients were sentinel node positive, of whom 52 patients were excluded due to protocol violations or patient preference and 474 were finally included; 244 ALND and and 230 aRT.  No. in trial arms: aRT: N = 230; ALND: N = 244  Age: aRT: Mean = 55.2 (range = 27-74) years; ALND: Mean = 54.7 (range = 26-74) years.  Stage distribution: aRT: pT1/pT2/pT3: N = 138/87/5; pN1ml/pN1a/pN2a/pN3a: N = NA. ALND: pT1/pT2/pT3: 105/123/16; pN1ml/pN1a/pN2a/pN3a: N = 61/129/41/13.  Proportion node positive: All were SL+.  Pathological type of breast cancer: aRT: Ductal: N = 188; lobular: N =28; other: N = 15. ALND: Ductal: N = 193; lobular: N = 40; other: N =11. | |
| **Interventions** | Women were randomised before surgery and SLNB to the treatment they would receive if their sentinel lymph node biopsy (SLNB) proved positive. Women with a positive lymph node received breast-conserving surgery or mastectomy and either axillary lymph node dissection (level I and II; at least 6 nodes; ALND ; undergone during primary surgery unless SLNs found to be positive solely by immunohistochemistry or HE staining in which case patients underwent ALND within 4-6 weeks) or axillary radiation therapy (within 8 weeks of surgery, including the contents of all three levels of the axilla and the supraclavicular fossa; 25 fractions of 2 Gy, 5 days per week; aRT) and radiotherapy to the remaining breast tissue and tumour bed (according to standard institutional protocols. | |
| **Outcomes** | Axillary recurrence, disease-free survival, overall survival | |
| **Axillary node surgery** | Minimum no. nodes to be removed according to protocol: ALND: at least 6 nodes.  Nodes removed aRT: Mean = 1.95 (range 1-5) nodes; mean number positive = 1.17 (range 1-4).  Nodes removed ALND: Mean = 14.31 (range 7-32) nodes; mean number positive = 2.77 (range 1-24).  Method of node pathological analysis: “serial sectioning (0.5-mm levels) and hematoxyline-eosin staining but no immunohistochemistry. All negative SLNs were investigated further by immunohistochemistry with a cytokeratin cocktail and epithelial membrane antigen.” | |
| **Radiotherapy** | ALND arm: Postoperative radiotherapy to the regional nodes to all patients with 4 or more positive nodes (pN2a-3a) and to patients with 1 to 3 positive nodes (pN1a) with other high-risk patient and tumour characteristics (eg, premenopausal status, lymphovascular invasion, histologic grade III tumor). 232/244 patients received radiotherapy to the breast/chest wall, 76/244 received radiotherapy to the axillary/supraclavicular nodes.  aRT: 208/230 patients received radiotherapy to the breast/chest wall, 230/230 received radiotherapy to the axillary/supraclavicular nodes. The proportions of patients receiving radiotherapy to the breast/chest wall did not differ significantly between the treatment arms, but significantly more aRT patients received radiotherapy to the axillary/supraclavicualr nodes (as per study design).  RT same in all trial arms? No | |
| **Hormone and chemotherapy** | “Adjuvant systemic therapies were administered according to our institutional protocols. Adjuvant trastuzumab treatment has been available since January 2008 at the National Institute of Oncology.”  ALND: 190/244 received chemotherapy; 213/244 received endocrine therapy; 6/244 received trastuzumab; 159/244 received chemotherapy and endocrine therapy.  aRT: 159/230 received chemotherapy; 204/230 received endocrine therapy; 13/230 received trastuzumab; 133/230 received chemotherapy and endocrine therapy. None of these proportions differed significantly between the treatment arms | |
| **Notes** | In 33 patients the sentinel node could not be identified. Partly published in Hungarian, which was translated by Laszlo Igali.  Baseline differences? Significantly more ALND (57%) than aRT (40%) patients had pT2-3 tumours.  Intention to treat analyses? Unclear | |
| **Bias** | **Authors' judgement** | **Support for judgement** |
| Random sequence generation (selection bias) | Unclear risk | No information reported |
| Allocation concealment (selection bias) | Unclear risk | No information reported |
| Blinding of outcome assessment (detection bias) Disease control in the axilla | Unclear risk | No information reported |
| Blinding of outcome assessment (detection bias) Breast cancer recurrence | Unclear risk | No information reported |
| Blinding of outcome assessment (detection bias) Short term adverse events | Unclear risk | No information reported |
| Blinding of outcome assessment (detection bias) Long term complications | Unclear risk | No information reported |
| Incomplete outcome data (attrition bias) Survival | Low risk | Data from all the patients were included in the analyses. |
| Incomplete outcome data (attrition bias) Disease control in the axilla | Unclear risk | Data not reported in sufficient detail to be able to ascertain whether all patients are included. |
| Incomplete outcome data (attrition bias) Breast cancer recurrence | Low risk | Data from all the patients were included in the analyses. |
| Incomplete outcome data (attrition bias) Short term adverse events | Unclear risk | Outcome not reported. |
| Incomplete outcome data (attrition bias) Long term complications | Unclear risk | Outcome not reported. |
| Selective reporting (reporting bias) | High risk | No morbidity outcomes reported |

**Ongoing studies**

**NCT01796444**

| **Study name** | [Axillary Lymph Node Dissection Versus no Dissection in Breast Cancer With Positive Sentinel Lymph Node](http://apps.who.int/trialsearch/Trial2.aspx?TrialID=NCT01796444) |
| --- | --- |
| **Methods** | Study design: RCT  Country: China |
| **Participants** | Inclusion criteria: Female, aged 18 years and above, histologically proven invasive breast cancer, clinical T1-T2 disease with no distant metastasis; clinical N0 status; patient for whom conservative surgery with sentinel lymph node (SLN) technique is feasible from the start in terms of carcinologic; Patient with positive SLNs 1~2; signed consent to participate.  Exclusion criteria: History of neoadjuvant chemotherapy or hormone therapy, history of breast cancer (ipsilateral, i.e. recurrence, or contralateral breast), history of any other invasive cancer, initial metastatic disease known, pregnant women or lactating women, impossibility to undergo medical examinations of the study for geographical, social or psychological reasons. |
| **Interventions** | ALND versus SLNB |
| **Outcomes** | Disease-free survival, overall survival, axillary recurrence rate |
| **Starting date** | 2013 |
| **Contact information** | Yong-sheng Wang, MD; Tel: +8613505409989; email: [wangysh2008@yahoo.com.cn](mailto:wangysh2008%40yahoo.com.cn?subject=NCT01796444,%20Z0011-China,%20Axillary%20Lymph%20Node%20Dissection%20Versus%20no%20Dissection%20in%20Breast%20Cancer%20With%20Positive%20Sentinel%20Lymph%20Node)  Peng-fei Qiu, Tel: MD +8615168872002; email: [qiupengfei2002@yahoo.cn](mailto:qiupengfei2002%40yahoo.cn?subject=NCT01796444,%20Z0011-China,%20Axillary%20Lymph%20Node%20Dissection%20Versus%20no%20Dissection%20in%20Breast%20Cancer%20With%20Positive%20Sentinel%20Lymph%20Node) |
| **Notes** | Other Study ID Numbers: Z0011-China |

**POSNOC**

| **Study name** | POSNOC: POsitive Sentinel NOde: adjuvant therapy alone versus adjuvant therapy plus Clearance or axillary radiotherapy. A randomised controlled trial of axillary treatment in women with early stage breast cancer who have metastases in one or two sentinel nodes. |
| --- | --- |
| **Methods** | Study design: RCT (multi-centre)  Country: United Kingdom |
| **Participants** | Inclusion criteria: "Women with unifocal or multifocal invasive breast cancer, largest primary lesion ≤ 5 cm, clinically and ultrasound node negative, who undergo sentinel node biopsy and have 1 or 2 sentinel node macrometastases (> 2mm), with no extranodal extension.  Exclusion criteria: Billateral breast cancer, more than 2 sentinel node macrometastases or extranodal invasion, neoadjuvant therapy for breast cancer, previous axillary surgery on the same body side as the scheduled sentinel lymph node biopsy, not fir or eligible to receive adjuvant systemic therapy, previous or concomitant malignancy except adequately treated basal or squamous cell carcinoma of the skin or adequately treated in situ carcinoma of the cervix or adequately treated in situ melanoma or contra- or ipsilateral in situ breast cancer. |
| **Interventions** | Adjuvant therapy alone versus adjuvant therapy plus axillary treatment (axillary node clearance or axillary radiotherapy) |
| **Outcomes** | Axillary recurrence at 5 years, local (breast or chest wall) and regional (nodal) recurrence, arm morbidity, quality of life, anxiety, distant metastasis, time to axillary recurrence, axillary recurrence free survival, disease free survival, overall survival, contralateral breast cancer, non-breast malignancy, economic evaluation. |
| **Starting date** | 2014 (projected publication date is 2024) |
| **Contact information** | Amit Goyal (Chief investigator), Royal Derby Hospital, Derby, UK; Tel: +44 1332785538/ +44 7588514469, email: amit.goyal@nhs.net. |
| **Notes** |  |
